# Acute benzyl alcohol intoxication: An autopsy case report

**DOI:** 10.1097/MD.0000000000033395

**Published:** 2023-03-31

**Authors:** Shojiro Ichimata, Yukiko Hata, Ryosuke Zaimoku, Naoki Nishida

**Affiliations:** a Department of Legal Medicine, Faculty of Medicine, University of Toyama, Toyama, Japan; b Department of Surgery, Kurobe City Hospital, Toyama, Japan.

**Keywords:** benzyl alcohol, central chromatolysis, grumose degeneration, intoxication, skin

## Abstract

The main mechanism of death and the pathological appearance of cases of benzyl alcohol intoxication has not been fully investigated. Autopsy reports of cases of benzyl alcohol intoxication have not been published.

**Case presentation:**

A 24-year-old man was found in the state of cardiopulmonary arrest at a construction site. He had been performing paint stripping. He was immediately transferred to the hospital, but he did not recover. An autopsy showed focal coloring of the skin without any major caustic injury. A histopathological investigation showed vacuolar degeneration in the epidermis and dermo-epidermal junction, and severe erosion of the tracheal and bronchial mucosa. No pathological changes in the kidney were evident. A neuropathological investigation showed central chromatolysis of neuronal cells in pontine nuclei and grumose degeneration in the cerebellar dentate nucleus. The blood content of benzyl alcohol was 780.0 μg/mL.

**Lessons:**

Present case suggest that multiple pathways of exposure may be associated with more rapid progression in acute benzyl alcohol intoxication, and that early and/or severe involvement of the central nervous system rather than renal dysfunction may be associated with an early death.

## 1. Introduction

Benzyl alcohol is a metabolite of toluene and is oxidized by an alcohol-depleting enzyme in the liver that produces benzoic acid.^[1,2]^ Benzoic acid is then combined with glycine and excreted in urine as hippuric acid.^[1]^ Benzyl alcohol is an ingredient in many paint removers and other industrial applications, and is even found in cosmetic products and food additives.^[2]^ There have been few case reports on benzyl alcohol intoxication in adults, and these cases showed impaired consciousness, respiratory depression, hypotension, metabolic acidosis, renal dysfunction, hypothermia, paralytic ileus, and hyperammonemia.^[2,3]^ However, autopsy reports of cases of benzyl alcohol intoxication have not been published. Therefore, the pathological appearance of cases of benzyl alcohol intoxication has not been fully investigated. We report a rare autopsy case of acute benzyl alcohol intoxication, and discuss the pathogenesis from the pathological appearance of this case.

## 2. Case presentation

A 24-year-old man was found collapsed at the workplace where he was painting. He had been performing paint stripping using a paint stripper containing benzyl alcohol to repaint a bridge of an express highway. The time interval between the final confirmation of the man healthy state and the discovery of his collapse was approximately 25 minutes. He suffered from cardiopulmonary arrest. He was transferred to the general hospital approximately 130 minutes after he was found, and manual cardiopulmonary resuscitation was performed up to arrival to the hospital. On arrival to the hospital, blood laboratory data showed that he was in a critical state because of higher concentrations of transaminases, enzymes of the musculature enzyme, and potassium (Table 1). Computed tomography showed no abnormal findings. He died 56 minutes after admission despite intensive resuscitation. He did not have any relevant clinical or family history.

**Table 1:** Laboratory findings at the time of admission.

Blood count (normal range)		
White blood cells	10,600	(3.5–9.1 × 10^3^)/μL
Red	4.7 × 10^6^	(3.7–5.0 × 10^6^)/μL
Hemoglobin	11.5	(11.3–15.2) g/dL
Hematocrit	37.6	(40–48) %
Platelet	41	(130–369 × 10^3^)/μL
Biochemistry (normal range)		
Aspartate transaminase	452	(10–40) IU/L
Alanine transaminase	391	(5–40) IU/L
Lactate dehydrogenase	1696	(115–359) IU/L
Alkaline phosphatase	374	(80–260) IU/L
Cholinesterase	327	(242–495) IU/L
r-GTP	35	(<70) IU/L
Creatinine kinase	319	(62–287) IU/L
CK-MB	153	(<25) IU/L
Total bilirubin	0.2	(0.2–1.2) mg/dL
Amylase	79	(34–125) IU/L
Total protein	7.6	(6.7–8.3) g/dL
Albumin	4.4	(3.8–5.2) g/dL
Urea nitrogen	13.0	(2.8–7.8) mmol/L
Uric acid	11.0	(3.0–7.0) mg/dL
Creatine	1.7	(<1.2) mg/dL
eGFR	43.6	(>90)
Sodium	139	(136–147) mmol/L
Potassium	13.8	(3.6–5.0) mmol/L
Chloride	103	(98–109) mmol/L
Calcium	9.6	(8.8–10.4) mmol/L
Inorganic phosphorus	14.0	(2.4–4.3) mmol/L
Magnesium	2.6	(0.74–1.07) mmol/L
Total cholesterol	194	(120–220) mg/dL
Triglyceride	39	(30–149) mg/dL
C-reactive protein	0.02	(<0.3) μg/L
Glucose	71	(80–99) mg/dL
Hemolysis	(++)	(−)

γ-GTP = γ-glutamyl transpeptidase, CK-MB = creatine kinase, myoglobin subtype, eGFR = estimated glomerular filtration rate.

**Figure 1.**
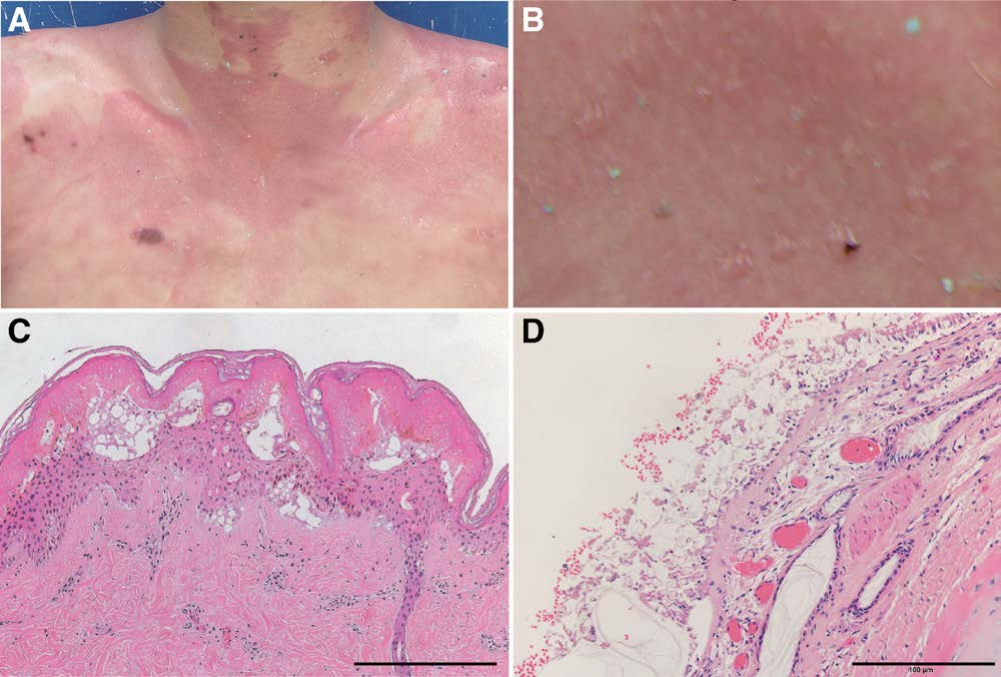
Gross and microscopic appearance of the skin and bronchus. (A) Reddish skin discoloration. (B) Bullous epidermolysis. (C) Vacuolar degeneration in the epidermis and dermo-epidermal junction (hematoxylin–eosin staining). (D) Degeneration of bronchial mucosa (hematoxylin–eosin staining). Scale bar = 200 mm (C) and 100 μm (D).

The postmortem interval at the time of autopsy was 12 hours. An autopsy showed discoloration of the skin with coating of an unknown material in the anterior neck, anterior chest, and both upper arms (Fig. 1A). Bullous epidermolysis was found on the discoloration area (Fig. 1B). No considerable macroscopic appearance was found in the internal organs other than congestion.

**Figure 2.**
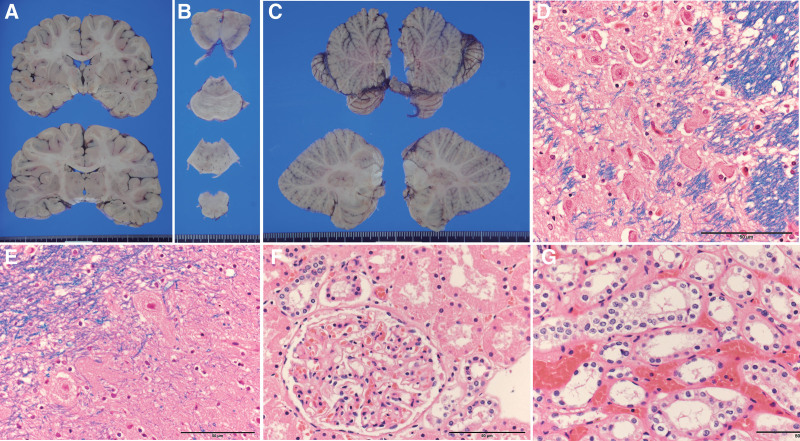
Pathological appearance of the brain, lung, and kidney. (A) Gross appearance of the cerebrum shows narrowing of the lateral ventricle due to edema. (B and C) The brain stem (B) and cerebellum (C) do not show any major pathological changes. (D) Central chromatolysis of neuronal cells in the pontine nucleus. (E) Grumose degeneration in the neurons of the cerebellar dentate nucleus. (F and G) A glomerulus and proximal renal tubules (F) and distal renal tubules (G) do not show any major pathological changes. (D and E) Luxol fast blue hematoxylin–eosin staining. (F and G) Hematoxylin–eosin staining. Scale bar = 200 μm (F), 100 μm (D), and 50 μm (E, G, H).

Microscopically, vacuolar degeneration in the epidermis and dermo-epidermal junction was observed (Fig. 1C). Degeneration of bronchial mucosa was also evident (Fig. 1D). The brain weighed 1602 g and showed marked edematous swelling, and also showed other pathological changes (Fig. 2A–C). A histopathological examination showed central chromatolysis in the neurons of the pontine nucleus, nucleus of the solitary tract, and nucleus ambiguus in the medulla oblongata (Fig. 2D). Additionally, grumose degeneration was observed in the cerebellar dentate nucleus (Fig. 2E). Amyloid precursor protein-positive axonal bulbs were not detected. Severe erosion with degeneration of the bronchial epithelium was evident (Fig. 2F), but degeneration of the tubular epithelium of the proximal and distal renal tubules was not found (Fig. 2G). The cardiac blood benzyl alcohol concentration was analyzed by headspace-gas chromatography-flame ionization detection methods (GC-2010, Shimadzu, Kyoto, Japan). Benzyl alcohol was quantified with a linear curve fit using methyl ethyl ketone as the internal standard. The blood benzyl alcohol concentration was 780.0 μg/mL.

## 3. Discussion

A lethal concentration of blood benzyl alcohol has not been established because of the rarity of such cases. Also, we should consider the possibility that a certain amount of postmortem diffusion from lung to cardiac blood may occur. However, the value in the present case was higher than that of a rescued, severe, symptomatic case (489 ± 5 μg/mL).^[4]^ Therefore, we consider that the cause of death in the present case was acute benzyl alcohol intoxication. To the best of our knowledge, this is the first autopsy report of a case of acute benzyl alcohol intoxication. Unfortunately, the present case was found in the state of cardiopulmonary arrest and died in a short interval. Therefore, the pathological appearance in the present case may have been caused by a direct effect of benzyl alcohol on the human body. A detailed pathological investigation may be useful for determining the possible pathogenesis leading to acute cardiopulmonary arrest in severe benzyl alcohol intoxication.

Inappropriate protective procedures might have been the cause of accidental intoxication of benzyl alcohol in the present case. Benzyl alcohol can be absorbed orally, by inhalation, or transdermally.^[1,2]^ Vacuolar degeneration in the epidermis and dermo-epidermal junction, which was found in the present case, may be consistent with macroscopic bullous epidermolysis. Vacuolar degeneration may be a sign of benzyl alcohol exposure as shown by a previous case report.^[2]^ Additionally, the degeneration of the bronchial mucosa may be pathological evidence of inhalation of benzyl alcohol in the present case. Exposure by inhalation and transdermal absorption may be associated with rapid progression of acute intoxication.

Benzyl alcohol is considered to cause direct injury to the central nervous system, but the specific mechanism and pathological appearance have not been fully established.^[5]^ McClosky et al reported a case of altered mental status after exposure to benzyl alcohol, and additionally showed that benzyl alcohol itself was toxic in a mouse model study.^[6]^ Experimental studies on the central neurotoxicity of toluene showed that multiple neurotransmitter and receptor systems, including N-methyl-D-aspartate receptors, dopamine neurons, and the g-aminobutyric acid transmission pathway, were affected by toluene exposure.^[7]^ Impairment of N-methyl-D-aspartate receptors, dopamine neurons, and the g-aminobutyric acid transmission pathway might occur in cases of acute benzyl alcohol intoxication. Central chromatolysis is a consequence of axonal injury, and chromatolysis can be characterized by reorganization of the cell soma and redistribution of Nissl substances to reconstitute injured axons.^[8]^ Grumose degeneration in the cerebellar dentate nucleus, which shows degeneration of the axon terminal of Purkinje cells,^[9]^ is found not only in chronic neurodegenerative disease (e.g., progressive supranuclear palsy and spinocerebellar degeneration), but also in acute ischemic/hypoxic encephalopathy.^[10]^ We recently found central chromatolysis and grumose degeneration in an autopsy case with rapid consciousness disturbance due to acute colchicine intoxication.^[11]^ The findings in the present case suggest that rapid progressive microscopic degeneration can occur in the central nervous system in a short interval after exposure to benzyl alcohol. Cardiac-projecting neurons of the nucleus ambiguus play a critical role in cardiac parasympathetic tone. Therefore, their activation elicits bradycardia via acetylcholine release in cardiac ganglia.^[12]^ Additionally, neurons in the nucleus of the solitary tract are essential for processing and coordinating respiratory and sympathetic responses to hypoxia.^[13]^ These studies indicate that pathological changes in the circulatory and respiratory centers in the medulla oblongata, as observed in the present case, may be strongly associated with the prognosis of acute benzyl alcohol intoxication.

Renal tubular dysfunction is considered to be a major complication, and associated symptoms are metabolic acidosis, hypokalemia, hypophosphatemia, and hyperammonemia.^[2,3]^ We could not evaluate renal tubular function and electrolyte values because the victim was already critical on admission. However, the pathological appearance of the kidney did not show degeneration of the tubular epithelium. The findings in the present case suggest that multiple pathways of exposure may be associated with more rapid progression in acute benzyl alcohol intoxication, and that early and/or severe involvement of the central nervous system may be associated with an early death.

## Acknowledgments

The authors thank Ms. Syuko Matsumori, Ms. Miyuki Maekawa, Ms. Misa Kusaba, and Mr. Osamu Yamamoto for their technical assistance. We thank Ellen Knapp, PhD, from Edanz (https://jp.edanz.com/ac) for editing a draft of this manuscript.

## Author contributions

**Conceptualization:** Shojiro Ichimata, Naoki Nishida.

**Data curation:** Shojiro Ichimata, Yukiko Hata, Ryosuke Zaimoku, Naoki Nishida.

**Investigation:** Shojiro Ichimata, Yukiko Hata, Ryosuke Zaimoku, Naoki Nishida.

**Supervision:** Ryosuke Zaimoku, Naoki Nishida.

**Validation:** Shojiro Ichimata, Yukiko Hata.

**Visualization:** Yukiko Hata.

**Writing – original draft:** Shojiro Ichimata.

**Writing – review & editing:** Naoki Nishida.
